# Comparative analysis of the neutralizing activity against SARS-CoV-2 Wuhan-Hu-1 strain and variants of concern: Performance evaluation of a pseudovirus-based neutralization assay

**DOI:** 10.3389/fimmu.2022.981693

**Published:** 2022-09-26

**Authors:** Luciana D’Apice, Maria Trovato, Giulia Gramigna, Francesca Colavita, Massimo Francalancia, Giulia Matusali, Silvia Meschi, Daniele Lapa, Aurora Bettini, Klizia Mizzoni, Luigi Aurisicchio, Antonino Di Caro, Concetta Castilletti, Piergiuseppe De Berardinis

**Affiliations:** ^1^ Institute of Biochemistry and Cell Biology, Consiglio Nazionale delle Ricerche (CNR), Naples, Italy; ^2^ National Institute for Infectious Diseases “L. Spallanzani” Istituto Ricovero e Cura a Carattere Scientifico (IRCCS), Rome, Italy; ^3^ Cancer Immunology, Takis Biotech, Rome, Italy

**Keywords:** COVID-19, SARS-CoV-2, variants of concern, pseudotyped virus, neutralization assay, neutralizing antibodies, vaccines, immunogenicity

## Abstract

**Objectives:**

Emergence of new variants of SARS-CoV-2 might affect vaccine efficacy. Therefore, assessing the capacity of sera to neutralize variants of concern (VOCs) in BSL-2 conditions will help evaluating the immune status of population following vaccination or infection.

**Methods:**

Pseudotyped viruses bearing SARS-CoV-2 spike protein from Wuhan-Hu-1/D614G strains (wild type, WT), B.1.617.2 (Delta), or B.1.1.529 (Omicron) VOCs were generated to assess the neutralizing antibodies (nAbs) activity by a pseudovirus-based neutralization assay (PVNA). PVNA performance was assessed in comparison to the micro-neutralization test (MNT) based on live viruses. Sera collected from COVID-19 convalescents and vaccinees receiving mRNA (BNT16b2 or mRNA-1273) or viral vector (AZD1222 or Ad26.COV2.S) vaccines were used to measure nAbs elicited by two-dose BNT16b2, mRNA-1273, AZD1222 or one-dose Ad26.CO2.S, at different times from completed vaccination, ~ 1.5 month and ~ 4-6 months. Sera from pre-pandemic and unvaccinated individuals were analyzed as controls. Neutralizing activity following booster vaccinations against VOCs was also determined.

**Results:**

PVNA titers correlated with the gold standard MNT assay, validating the reliability of PVNA. Sera analyzed late from the second dose showed a reduced neutralization activity compared to sera collected earlier. Ad26.CO2.S vaccination led to very low or absent nAbs. Neutralization of Delta and Omicron BA.1 VOCs showed significant reduction of nAbs respect to WT strain. Importantly, booster doses enhanced Omicron BA.1 nAbs, with persistent levels at 3 months from boosting.

**Conclusions:**

PVNA is a reliable tool for assessing anti-SARS-CoV-2 nAbs helping the establishment of a correlate of protection and the management of vaccination strategies.

## Introduction

Levels of neutralizing antibodies (nAbs) are highly predictive of immune protection from symptomatic SARS-CoV-2 infection and thereby are strong predictors of vaccine efficacy (1, 2). Micro-neutralization test (MNT) is the gold standard assay for detecting nAbs. Nevertheless, the assay poses biosafety concerns related to the use of live viruses that limit its availability to a restricted number of high-containment laboratories. To overcome these issues, several pseudovirus-based neutralization assays (PVNAs) have been proposed and used worldwide (3–5).

In the effort of curtailing the persistent spread of severe acute respiratory syndrome coronavirus 2 (SARS-CoV-2), eleven vaccines (as of June, 2022) have been authorized by the World Health Organization (WHO) for full or emergency use (https://covid19.trackvaccines.org/agency/who/). The approved vaccines exploit four distinct technological platforms and comprise the mRNA-based BNT16b2 (Pfizer/BioNtech) and mRNA-1273 (Moderna), and the non-replicating adenoviral vector Ad26.COV2.S (Johnson & Johnson/Janssen) and AZD1222 (ChAdOx1 vector, AstraZeneca/University of Oxford) vaccines. These vaccines were designed to deliver (or express) the full-length SARS-CoV-2 spike (S) protein derived from the ancestral Wuhan-Hu-1 strain and have been extremely effective in preventing severe coronavirus disease 2019 (COVID-19) (6). Nevertheless, COVID-19 breakthrough infections have been reported in vaccine recipients, likely due to waning immunity and/or emergence of viral variants escaping vaccine-induced immunity (7, 8).

B.1.617.2 (Delta; first identified in India) lineage of SARS-CoV-2 became the predominant circulating variant at the beginning of 2021, quickly followed by the higher transmissible B.1.1.529 (Omicron, first reported from South Africa; BA.1 sublineage) variant (9) in November 2021, and were subsequently defined as variants of concerns (VOCs), carrying S polymorphisms.

Several studies showed a reduced *in vitro* neutralization of the circulating VOC by sera from people who are infected with the ancestral strains or immunized with authorized vaccines (10).

The aim of this study was to evaluate performance and reliability of a PVNA protocol in comparison to the gold standard based on live virus in the detection of nAb response against SARS-CoV-2 Wuhan-Hu-1/D614G strains (wild type, WT), Delta and Omicron BA.1 variants in residual anonymized serum samples collected from unvaccinated individuals, COVID-19 convalescents and vaccinees early and late from second vaccine dose administration. Finally, effectiveness of booster shots at enhancing vaccinees’ immunity was investigated.

## Materials and methods

### Study design and participants

Residual serum samples stored following the diagnostic routine activities performed at the National Institute for Infectious Diseases “L. Spallanzani” (INMI, Rome, Italy) were anonymized and used to test and validate the performance of the novel PVNA as compared to the results obtained with MNT, the gold standard based on live virus. Cohorts, time points of analysis and sample characteristics are described in **Supplementary Methods**.

The use of residual anonymized samples for the validation of new diagnostic tools and the longitudinal collection for the monitoring of antibody response induced by the vaccination were approved by the “Comitato Etico INMI Lazzaro Spallanzani IRCCS/Comitato Etico Unico Nazionale Covid-19” (issues n. 70/2018 and n. 55/2022, respectively).

### Anti-SARS-CoV-2 IgG chemiluminescence microparticle assay

As previously described (11), to detect anti-Nucleoprotein (anti-N) IgG and anti-Spike/RBD IgG, the following assays were used: ARCHITECT SARS-CoV-2 IgG, and ARCHITECT SARS-CoV-2 IgG II Quantitative, on ARCHITECT^®^ i2000sr; Abbott Laboratories, Wiesbaden, Germany. According to the manufacturer’s instructions, Index >1.4 and Binding Antibody Units (BAU)/mL ≥7.1 are considered positive for anti-N and anti-Spike/RBD IgG, respectively. According to the WHO standard preparation for SARS-CoV-2 binding antibodies (12), the results expressed as BAU/ml were obtained using the following conversion factor from Abbott AU: 1 BAU/mL = 0.142 × AU/mL.

### Micro-neutralization test

nAbs were assessed by micro-neutralization test (MNT) using live SARS-CoV-2 viruses in BSL-3 facility. The challenging viruses included (i) the Wuhan-D614G strain, isolated in March 2020 in Italy (GISAID accession ID EPI_ISL_ 568579; Ref-SKU: 008V-04005, from EVAg portal), (ii) the Delta strain (GISAID accession ID EPI_ISL_3230211) and (iii) the Omicron BA.1 strain (GISAID accession ID EPI_ISL_7716384), isolated from a traveler who arrived to Italy in December 2021. Viral stocks were prepared by infecting Vero E6 cells and titre calculated according to the Reed and Muench method and expressed as 50% Tissue Culture Infective Dose (TCID50/ml) (13). For MNT, seven two-fold serial dilutions in cell medium containing 2% fetal bovine serum (starting dilution 1:10) of heat-inactivated serum samples (56°C for 30 min) were titrated in duplicate, mixed with the same volume (50 μl) containing 100 TCID50 SARS-CoV-2 and incubated at 37°C for 30 min. Subsequently, virus-serum mixtures were added to sub-confluent Vero E6 cells seeded in 96-well microplates and incubated at 37 C, 5% CO2. Microplates were observed by light microscope for the presence of cytopathic effect (CPE) after 48 hours for the Wuhan-D614G strain and after 96 hours for the Omicron and Delta strains. Neutralization titres were expressed as the reciprocal of the highest serum dilution inhibiting at least 90% (MNT_90_) of CPE. When 90% inhibition was not observed at the first dilution tested (1:10), the sample was considered not able to neutralize (neutralization titre <10). To standardize the inter-assay procedures, positive control samples showing low (20) and high (160) neutralizing activity were included in each session. In addition, serum from the National Institute for Biological Standards and Control, Blanche Lane, Ridge, Herts, UK (NIBSC) with known neutralization titer (Research reagent for anti-SARS-CoV-2 Ab NIBSC code 20/136) was used as a reference in MNT during the setting-up ad validation of the protocol.

### Production and titration of SARS-CoV-2 S-pseudotyped lentiviruses

SARS-CoV-2 S-pseudotyped lentiviral particles harboring the Luciferase (Luc) reporter gene were produced by transfection of HEK293T cells (seeded 24 hours before transfection at a density of 3.5 × 10^6^ cells/100 mm Petri dish) (14–16), as reported in **Supplementary Methods**. The neutralization activity of each serum sample was calculated as follows: % Neutralization = (RLU_max_ – RLU_experimental_)/(RLU_max_ – RLU_min_)*100, where RLU_max_ was the maximal infectivity calculated from untreated infected cells, RLU_experimental_ was calculated from infected cells treated with each serum dilution, RLU_min_ was the minimal infectivity calculated from uninfected cells (17). The nAb titers were expressed as the reciprocal of the highest serum dilution leading to 90% inhibition of RLUs (IC_90_). All samples with neutralization titers < 10 were considered negative and given an arbitrary value of IC_90_ = 5. The highest serum dilutions resulting in 90% or 50% reduction of luciferase production were referred to as pVNT_90_ or pVNT_50_, respectively. Neutralization of VSVg pseudotyped lentivirus infection was used to assess false positive results.

### Statistical analysis

Statistical analysis was performed using GraphPad Prism 9 (GraphPad Software, Inc., San Diego, CA). Spearman rank correlation was used as non-parametric test to measure the association between 90% live SARS-CoV-2 neutralization titers (MNT_90_) and 90% pseudovirus neutralization titers (pVNT_90_). Ratio between pVNT_90_ mean titers and MNT_90_ mean titers (pVNT_90_/MNT_90_) was also calculated and included on correlation plots to provide the mean bias between PVNA and MNT. The Spearman’s rank correlation coefficients r_s_ and the *p* values in serum samples from all study participants are presented in **Table S1**. Kruskal–Wallis test with Dunn’s multiple comparison post-test was used to compare the nAb titers among vaccine groups. A two-tailed non-parametric Wilcoxon signed-rank test for paired observations or a two-tailed non-parametric Mann–Whitney test for unpaired observations was performed. *P*-values < 0.05 were considered to be statistically significant. * p < 0.05; ** p ≤ 0.01; *** p ≤ 0.001; **** p < 0.0001. Concordance between the two assays and k coefficient of agreement were also established.

## Results

### Cohort description and binding antibody responses to SARS-CoV-2

**Figure 1:**
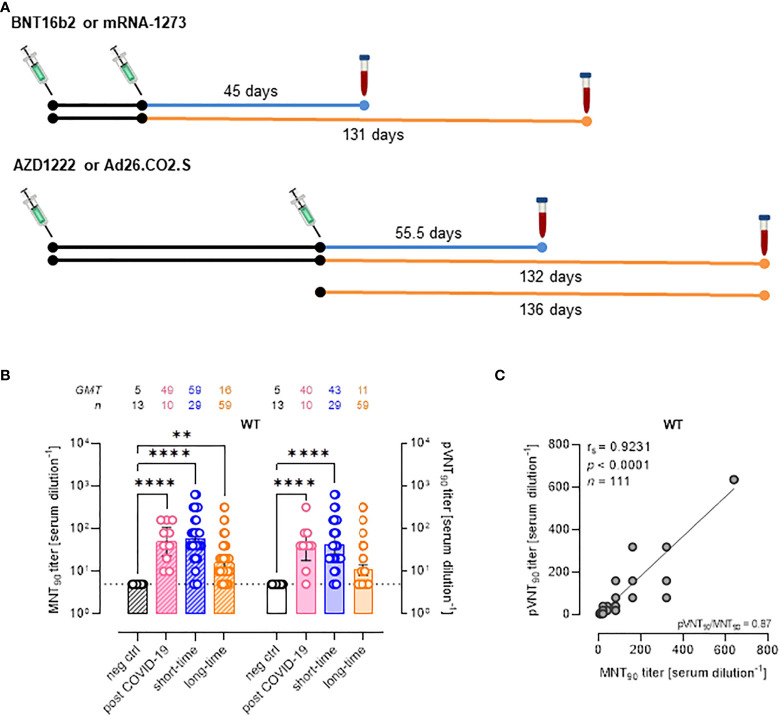
Serum collection schedule and nAb antibody detection. **(A)** Schematic representation of time points (blue: short-time; orange: long-time) of serum collection for each cohort of donors. The median time from the 2nd dose of BNT162b2, mRNA-1273, and AZD1222 was of 56 days (range 20–68 days), 44.5 days (range 22–67), and 55.5 days (range 20–72), respectively. The median time from the 2nd dose of BNT162b2, mRNA-1273, AZD1222, and from the 1st dose of Ad26.CO2.S was of 138 days (range 101–191 days), 128 days (range 110–172), 132 days (range 110–192), and 136 days (range 120–162), respectively. **(B)** 90% live SARS-CoV-2 wild type (WT) neutralization titers (MNT_90_, left y-axis, hatched bars) and 90% pseudovirus neutralization titers (pVNT_90_, right y-axis, open bars) were used as measure of nAb levels in sera from SARS-CoV-2 infected recovered donors or sera from vaccinated donors collected at different time points. Geometric means ± 95% confidence intervals were reported. Neutralization titers < 10 were considered negative and given an arbitrary value of 5 (dotted line). Kruskal–Wallis test with Dunn’s multiple comparison post-test was used to compare groups. ** *p* ≤ 0.01; **** *p* < 0.0001. **(C)** Correlation between pVNT_90_ and MNT_90_ titers against SARS-CoV-2 WT strain. The non-parametric Spearman’s correlation coefficients (r_s_), statistically significant *p* values and ratio between pVNT_90_ mean titers and MNT_90_ mean titers were provided. Perfect-fit correlation line was included on the plot. *GMT*: geometric mean titers; *n* = number of samples.

Samples negative for anti-SARS-CoV-2 were used as negative controls for the assessment of the assay. These samples included pre-pandemic sera collected in early 2019 and unvaccinated healthy individuals who showed absence of anti-N and anti-Spike/RBD IgG. These groups included 7 females and 7 males, with a mean age of 51 ± 17 years (median:51; IQR:36.7–59.2). Samples from COVID-19 convalescent individuals collected at a median time of 93 days (interquartile range, IRQ=84.5-99) from the infection were positive to anti-Spike/RBD IgG with median levels of 397 BAU/mL (IQR: 168.9-574.9 BAU/mL). This group included 4 females and 6 males, with a mean age of 61 ± 10 years (median:58; IQR:53.5-63). Samples collected from vaccinees were selected according two different time points from the last vaccine dose administration (**Figure 1A**): “short-time” consisting in samples collected after ~ 1.5 mo from the second vaccine dose and “long-time” consisting in samples collected after 4-6 mo from the last vaccine dose administration. These two groups included 43 females and 46 males, respectively, with a mean age of 58 ± 15 years (median:56; range:49.8–70.7). In addition, although this study did not aim to compare vaccine formulations, in order to evaluate the assay performance in a more realistic population, we selected samples from individuals receiving different vaccines, BNT162b2, mRNA-1273, AZD1222, Ad26.COV2.S, with no differences in age and gender. All vaccinees were negative to anti-N IgG and presented positive anti-Spike/RBD IgG both at short and long time from last dose vaccine (**Figure S1**). Finally, the cohort of vaccinees receiving booster dose of mRNA vaccine included 14 females and 4 males with a mean age of 46 ± 13 years (median:48; IQR:36-56).

### SARS-CoV-2 pseudotyped system efficiently detects neutralizing antibodies: Comparison with the MNT reference test

The PVNA, using SARS-CoV-2 pseudoviruses carrying the WT SΔ19 protein, was first compared to the MNT using live SARS-CoV-2 Wuhan-Hu-1/D614G strains (wild type, WT). Results were measured as pVNT_90_ and MNT_90_ titers, respectively. Of the 14 samples negative for anti-SARS-CoV-2 antibodies, all resulted negative also for nAbs using MNT, while PVNA detected correctly 13 (93.0%) samples as negative. The sample with false positive result (pVNT_90 =_ 1:160) showed reactivity also against the VSVg pseudotyped lentivirus, thus it was excluded from further analysis.

The reliability of SARS-CoV-2 pseudotyped system in detecting nAbs was validated using samples positive for anti-Spike/RBD IgG collected from a cohort of COVID-19 convalescent individuals (n=10) collected after a median time of 93 days from symptoms onset, and from a cohort of vaccinees (n=89) at different time points from the last dose administration (**Figure 1A**). Among 99 anti-Spike/RBD IgG positive samples, 83 (83.8%) had positive nAbs by MNT (MNT90≥1:10); of these samples, PVNA detected nAbs in 64 (77.1%) samples. One of these samples showed cross-reactivity against the VSVg pseudotyped lentivirus, resulting with pVNT90 = 1:160 vs MNT_90 =_ 1:20. Nineteen samples resulted positive for nAbs in MNT but negative in PVNA. Notably, false-negative results by PVNA were observed in those samples with low MNT_90_ (between 1:10 and 1:20).

Overall, considering the total of the samples (n=113) tested against WT SARS-CoV-2, the comparison between the two assays showed a “substantial agreement” for nAb detection (82.3% concordance, k coefficient = 0.619; 95%CI: 0.477-0.761). The result was confirmed also when excluding the two samples showing reactivity also against the VSVg pseudotyped lentivirus (82.9% concordance, k coefficient = 0.634; 95%CI: 0.495-0.773). In addition, similar results in nAb titres were observed, as confirmed also by the Spearman’s rank correlation (r_s_ = 0.9231; p < 0.0001) (**Figures 1B, C** **)**.

**Figure 2:**
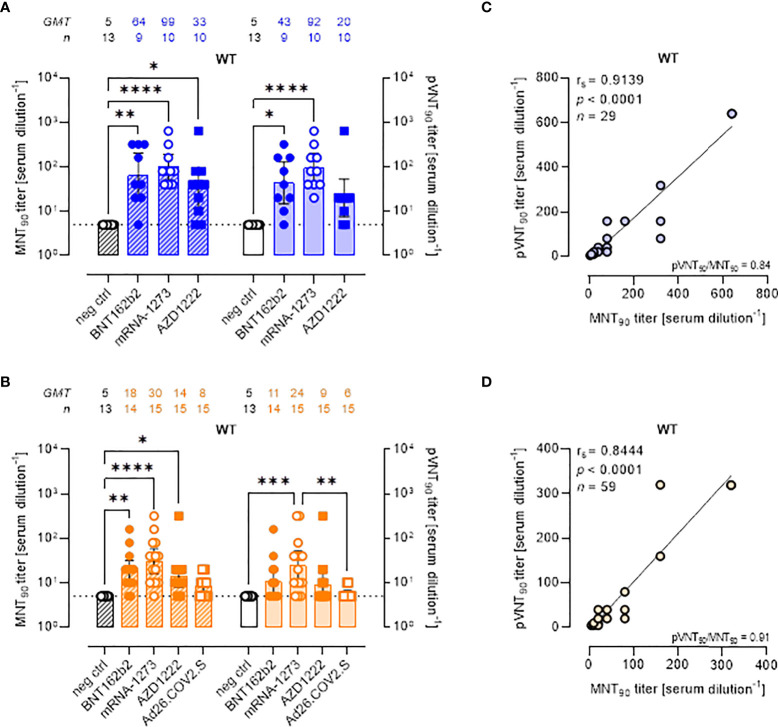
Live virus MNT and SARS-CoV-2 PVNA on sera collected from vaccinated donors produce comparable results. **(A, B)** 90% live SARS-CoV-2 wild type (WT) neutralization titers (MNT_90_, left y-axis, hatched bars) and 90% pseudovirus neutralization titers using pseudotyped lentiviruses carrying the WT SΔ19 protein (pVNT_90_, right y-axis, open bars) in sera drawn from **(A)** unvaccinated and recipients of two-dose BNT162b2, mRNA-1273, and AZD1222 collected at a median time of ~ 1.5 mo post-second vaccine dose; **(B)** unvaccinated and recipients of two-dose BNT162b2, mRNA-1273, AZD1222, or one dose Ad26.CO2.S collected at a median time of ~ 4–6 mo post-vaccination. Geometric means ± 95% confidence intervals were reported. Neutralization titers < 10 were considered negative and given an arbitrary value of 5 (dotted line). Kruskal–Wallis test with Dunn’s multiple comparison post-test was used to compare groups. * *p* < 0.05; ** *p* ≤ 0.01; *** *p* ≤ 0.001; **** *p* < 0.0001. **(C)** Correlation between pVNT_90_ and MNT_90_ titers shown in **(A)**; **(D)** correlation between pVNT_90_ and MNT_90_ titers shown in **(B)**. The non-parametric Spearman’s correlation coefficients (r_s_), statistically significant *p* values and ratio between pVNT_90_ mean titers and MNT_90_ mean titers were provided. Perfect-fit correlation line was included on the plots. *GMT*: geometric mean titers; *n* = number of samples.

Although the aim of the work was not to compare vaccine platforms, serum samples from vaccinated volunteers were analyzed in detail comparing the two assays (**Figure 2**). Both the MNT and PVNA assays revealed neutralizing activity at short time points (~ 1.5 mo) post-second vaccine dose in a total of 26/29 (89.7%) samples, consisting in 88.9% (8 of 9) of individuals receiving BNT162b2, 80.0% (8 of 10) receiving AZD1222 and 100% (10 of 10) of mRNA-1273 vaccinees (**Figure 2A**). When sera collected at longer time points (4–6 mo) were analyzed, a reduction of nAb levels were observed and were detected in 78.3% (47 of 60) and in 48.3% (29 of 60) of vaccinees by MNT or PVNA, respectively (**Figure 2B**). Notably, for this time point, we included in the study also sera collected from donors vaccinated with Ad26.COV2.S (which is administered as single dose). In this latter case, very low or absent nAb levels were observed (**Figure 2B**).

Comparing the nAb titres, the MNT and the PVNA assays showed very similar GMTs (**Figures 2A, B**). Indeed, pVNT_90_ titers strongly correlated with MNT_90_ titers across samples collected both early (**Figure 2C**, r_s_ **=** 0.9139) and late (**Figure 2D**, r_s_ = 0.8444) from vaccination, validating the reliable performance of SARS-CoV-2 pseudotyped system. Correlation analyses revealed also a positive association between MNT_90_ titers and levels of IgG to RBD (**Figure S2A**, r_s_ **=** 0.8429; **Figure S2B**, r_s_ **=** 0.8215) and between pVNT_90_ titers and anti-RBD IgG (**Figure S2C**, r_s_ **=** 0.8843; **Figure S2D**, r_s_ **=** 0.7953) across all analyzed samples. The highest correlation was observed between pVNT_90_ titers and anti-RBD IgG levels in sera collected early from vaccination (**Figure S2C**, r_s_ **=** 0.8843).

When SARS-CoV-2 WT neutralization was analyzed at a threshold of 50% reduction of Luc production (pVNT_50_), as expected, an increase of GMTs was observed in each study cohort, except for pre-pandemic and unvaccinated not infected individuals (neg ctrl) (**Figures S3A-C**). The highest GMT values were observed in sera collected at short- or long-time from mRNA-1273 vaccination (**Figures S3B, C**), consistent with the 90% neutralization analysis.

### PVNA vs MNT for Delta and Omicron variants: Neutralization activity is impaired by mutations in VOCs

**Figure 3:**
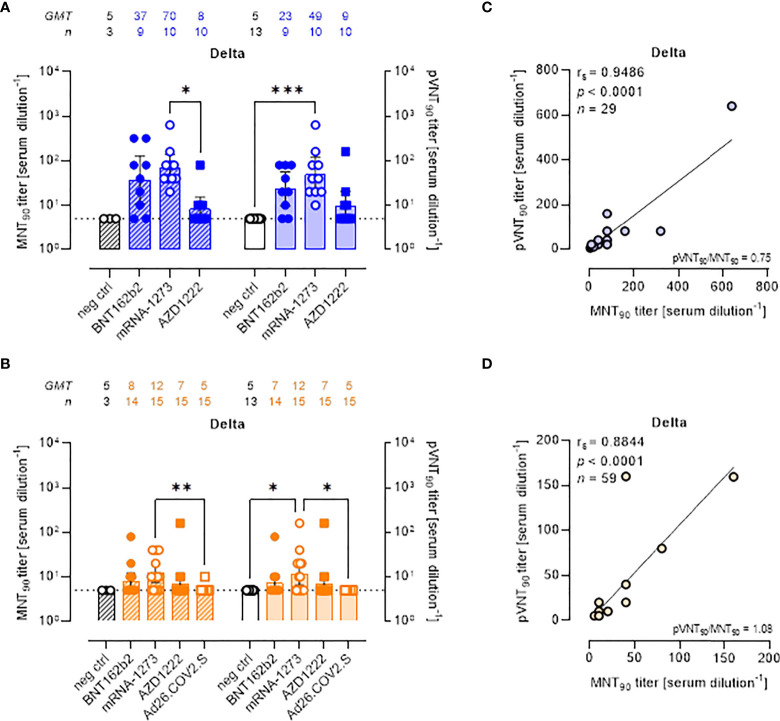
Neutralizing antibodies against SARS-CoV-2 Delta variant are detected either by MNT or PVNA. **(A, B)** 90% live SARS-CoV-2 Delta neutralization titers (MNT_90_, left y-axis, hatched bars) and 90% pseudovirus neutralization titers using pseudotyped lentiviruses carrying the Delta SΔ19 protein (pVNT_90_, right y-axis, open bars) in sera drawn from **(A)** unvaccinated and recipients of two-dose BNT162b2, mRNA-1273, and AZD1222 collected at a median time of ~ 1.5 mo post-second vaccine dose; **(B)** unvaccinated and recipients of two-dose BNT162b2, mRNA-1273, AZD1222, or one dose Ad26.CO2.S collected at a median time of ~ 4–6 mo post-vaccination. Geometric means ± 95% confidence intervals were reported. Neutralization titers < 10 were considered negative and given an arbitrary value of 5 (dotted line). Kruskal–Wallis test with Dunn’s multiple comparison post-test was used to compare groups. * *p* < 0.05; ** *p* ≤ 0.01; *** *p* ≤ 0.001. **(C)** Correlation between pVNT_90_ and MNT_90_ titers shown in **(A)**; **(D)** correlation between pVNT_90_ and MNT_90_ titers shown in **(B)**. The non-parametric Spearman’s correlation coefficients (r_s_), statistically significant *p* values and ratio between pVNT_90_ mean titers and MNT_90_ mean titers were provided. Perfect-fit correlation line was included on the plots. *GMT*: geometric mean titers; *n* = number of samples.

**Figure 4:**
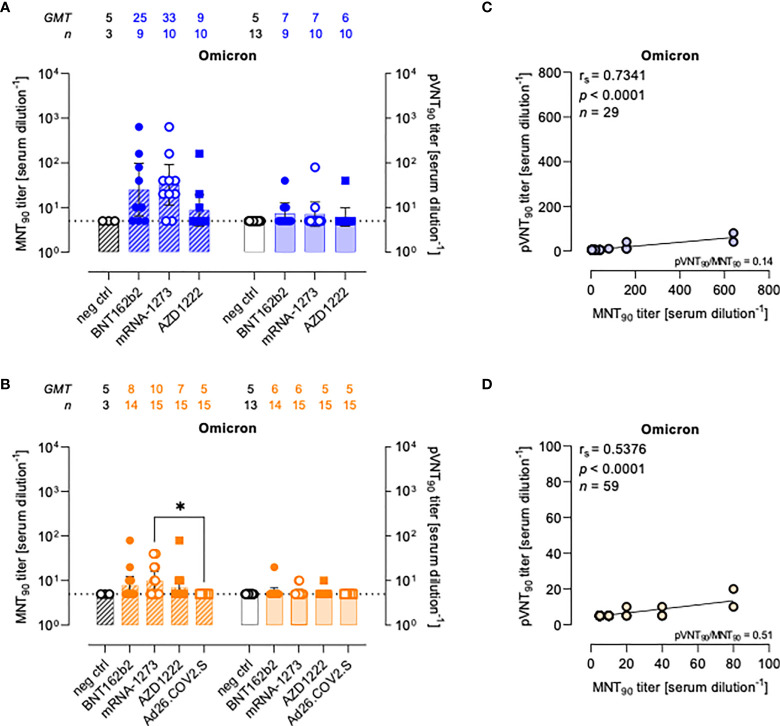
Neutralizing antibodies against SARS-CoV-2 Omicron variant are detected either by MNT or PVNA. **(A, B)** 90% live SARS-CoV-2 Omicron neutralization titers (MNT_90_, left y-axis, hatched bars) and 90% pseudovirus neutralization titers using pseudotyped lentiviruses carrying the Omicron SΔ19 protein (pVNT_90_, right y-axis, open bars) in sera drawn from **(A)** unvaccinated and recipients of two-dose BNT162b2, mRNA-1273, and AZD1222 collected at a median time of ~ 1.5 mo post-second vaccine dose; **(B)** unvaccinated and recipients of two-dose BNT162b2, mRNA-1273, AZD1222, or one dose Ad26.CO2.S collected at a median time of ~ 4–6 mo post-vaccination. Geometric means ± 95% confidence intervals were reported. Neutralization titers < 10 were considered negative and given an arbitrary value of 5 (dotted line). Kruskal–Wallis test with Dunn’s multiple comparison post-test was used to compare vaccine groups. * *p* < 0.05. **(C)** Correlation between pVNT_90_ and MNT_90_ titers shown in **(A)**; **(D)** correlation between pVNT_90_ and MNT_90_ titers shown in **(B)**. The non-parametric Spearman’s correlation coefficients (r_s_), statistically significant *p* values and ratio between pVNT_90_ mean titers and MNT_90_ mean titers were provided. Perfect-fit correlation line was included on the plots. *GMT*: geometric mean titers; *n* = number of samples.

All approved COVID-19 vaccines were developed against the ancestral S protein. Studies evaluating the effectiveness of different vaccines are required to understand the potential ability of VOCs to escape from vaccine-induced immunity. To evaluate the neutralization activity against infection by VOCs, the two neutralization assays were performed using live SARS-CoV-2 Delta and Omicron BA.1 variants and pseudoviruses carrying the Delta and Omicron BA.1 SΔ19 proteins. MNT_90_ and pVNT_90_ titers to Delta and Omicron VOCs were then compared (**Figures 3**, **4**). The MNT_90_ titers in all vaccinated individuals were lower against the Delta variant (**Figures 3A, B** **)** than against the WT strain (**Figures 2A, B**). The highest GMT values were observed in sera collected at short time from mRNA-1273 vaccination (**Figure 3A**). Sera collected late from vaccination showed a reduced neutralizing activity compared to those collected early, with the mRNA-1273 vaccinees having higher Delta nAbs (**Figure 3B**). Similar results were observed when sera were analyzed by PVNA assay, as confirmed by the positive correlations shown in **Figure 3C** (r_s_ **=** 0.9486, p<0.0001) and **Figure 3D** (r_s_ **=** 0.8844, p<0.0001). The 50% neutralization analysis gave comparable results, with the mRNA-1273 group having the highest Delta GMTs (**Figures S4A, B**).

There was a reduction in the neutralizing activity against the Delta variant as compared to WT in all sera collected early and late from vaccination with two doses of BNT162b2 (**Figures S5A, B**), mRNA-1273 (**Figures S5C, D**), and AZD122 (**Figures S5E, F**). None of sera collected late from one-dose Ad26.COV2.S vaccination had detectable nAbs against the Delta variant (**Figure S5G**).

Compared to WT neutralization (**Figure 2**), neutralization activities against Omicron VOC resulted impaired in all vaccinated individuals with both assays (**Figures 4A, B**), for which the MNT_90_ and pVNT_90_ titers positively correlated as shown in **Figures 4C, D**. Additional analyses using sera from COVID-19 convalescents demonstrated an impairment of the neutralizing activity against Omicron VOC also in this cohort (**Figure S6**).

The pVNT_90_ titers reported in **Figure S7** points out how the mutation accumulated in the S protein of the Omicron BA.1 variant affects the neutralizing activity induced by the different vaccines either at short or at long time since completing the vaccination schedule. Relative to GMT values against WT, in line with the general waning of the immune response, there was respectively a 6.1-fold and 1.8-fold reduction early and late from vaccination with two doses of BNT162b2 (**Figures S7A, B**), a 13.1-fold and 4-fold reduction early and late from vaccination with two doses of mRNA-1273 (**Figures S7C, D**), and a 3.3-fold and 1.8-fold reduction early and late from vaccination with two doses of AZD1222 (**Figures S7E, F**). None of sera collected late from the single-dose Ad26.COV2.S vaccination had detectable nAbs against the Omicron variant for both assays (**Figure S7G**).

### Booster shots increase nAbs against WT and Omicron variant

**Figure 5:**
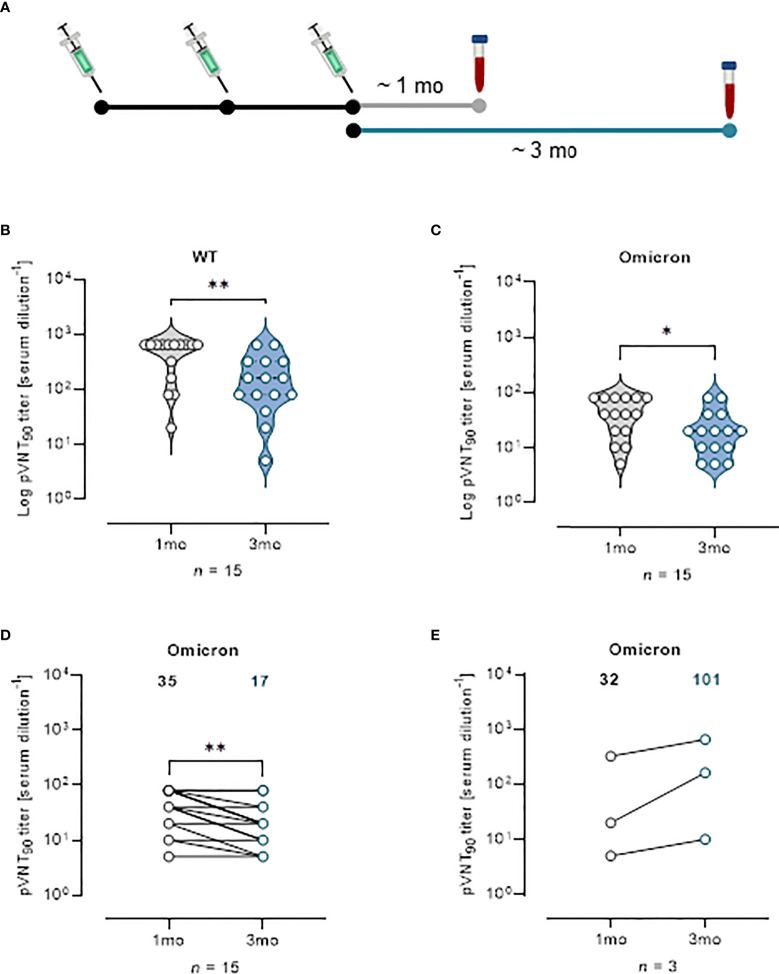
Vaccine booster shots strengthen nAb response against Omicron VOC. **(A)** Schematic representation of serum collection time points. **(B, C)** Violin plots representing Log of pVNT_90_ observed in sera (*n* = 15) collected at ~ 1 mo or ~ 3 mo after vaccine boost and challenged with **(B)** SARS-CoV-2 WT or **(C)** Omicron pseudotyped lentiviruses. A two-tailed non-parametric Mann–Whitney test for unpaired observations was performed. * *p* < 0.05; ** p ≤ 0.01. **(D)** pVNT_90_ titers against Omicron VOC in sera collected at ~ 1 mo and ~ 3 mo after vaccine boost report immune response waning after three months. ** p ≤ 0.01. **(E)** pVNT90 titers against Omicron VOC observed in sera from three donors infected with SARS-CoV-2 and excluded from the previous analyses. A two-tailed non-parametric Wilcoxon signed-rank test was performed for paired observations **(D, E)**. Geometric mean titers are reported. *n* = number of samples.

Effect of vaccine boosters at enhancing protection against VOCs was investigated analyzing sera from 18 mRNA recipients collected at a median time of ~ 1 mo, (26 days) and ~ 3 mo (96 days) from the third vaccine dose (**Figure 5A**). The pVNT_90_ titers to SARS-CoV-2 WT strain or Omicron BA.1 variant in this cohort are reported in **Figures 5B, C**, respectively. Boosted vaccinees had a WT GMT of 335.1 at ~ 1 mo after the third dose, that significantly decreased to a GMT of 115.8 at ~ 3 mo from boosting (**Figure 5B**). The Omicron GMT at ~ 1 mo post-third vaccine dose was of 35 that significantly decreased to a GMT of 17 following ~ 3 mo from the booster shots (**Figure 5C**). It should be emphasized that after vaccine boosting (regardless of the type of vaccine previously administered), nAb GMT against the Omicron VOC increased in comparison to the results observed for individuals receiving two doses of vaccine (**Figures 3**, **4**). In **Figure 5D** the correlative statistical analysis for paired samples was reported to compare the individual response against Omicron at ~ 1 mo and ~ 3 mo from boosting. Although a significant reduction of the neutralizing activity was observed (fold decrease of 2.1), boosted vaccinees had persistent levels of Omicron nAbs after 3 months from boosting. In the interval that occurred between the blood sampling, 3 donors were infected by SARS-CoV-2 and were excluded from the analysis. Individuals exposed to the virus had a GMT of 32 that increased to 101 following the infection (**Figure 5E**), consistent with finding previously reported (18). When neutralization was analyzed at a threshold of 50%, WT GMTs decreased from 442.2 at ~ 1 mo after the third dose to a GMT of 291.8 at ~ 3 mo from boosting (**Figure S8A**). The Omicron GMT at ~ 1 mo post-third vaccine dose was of 87.8 that significantly decreased to a GMT of 33.3 following ~ 3 mo from the booster shots (**Figure S8B**).

## Discussion

Understanding the quantity, quality and duration of the antibody response is compelling to predict protection against SARS-CoV-2. It has been demonstrated that assessing the level of nAbs that block viral entry into cells is a fundamental parameter in determining protection (1). However, with the ongoing of COVID-19 epidemic, new strains of SARS-CoV-2 (VOCs) are continuously emerging. These variants quickly spread worldwide and caused the four waves of global epidemics. Evaluation of the efficacy of the antibody response, either following vaccination or natural infection, against different VOCs is of capital interest in the attempt of estimating how emerging variants could be recognized and eventually evade the immune response in the population. Moreover, the availability of standardized and easy-to-implement methods for measuring nAbs could in the future allow a more personalized approach to vaccination strategies.

Determining neutralization activity poses challenges, as these assays generally rely on live virus replication, requiring a high containment (BSL-3) laboratory, specialized personnel and well-established protocols from the virus isolation and culture to the neutralization assay. To address this issue, a variety of surrogate assays have been proposed, based on enzyme-linked immunosorbent assay (ELISA) test or on live surrogate virus, such as live attenuated SARS-CoV-2 or pseudoviruses (3, 19–24). ELISA-based test presents advantages such as low cost, speed, and safety but only antibodies that block the RBD/ACE2 interaction are detected, thus both the neutralizing activity and the detection of non-RBD binding antibodies which may also be neutralizing are missing. Live attenuated and replication-deficient pseudotyped-virus neutralization assays are a safe alternative to evaluate protective antibody response in sera from vaccinees or convalescent individuals. The properties of the engineered viruses allow nAb experiments to be performed in BSL-2 laboratories. This is of a great advantage, as highly pathogenic viruses such as SARS-CoV-2 require BSL-3 laboratories. Different pseudovirus-based assays have been reported and widely used for the detection of anti-SARS-CoV-2 nAbs with the potential to be deployed for the large-scale testing as high-throughput screening of COVID-19 patients or vaccinated people in general lab settings (25). Live attenuated SARS-CoV-2 pseudoviruses are more similar to the authentic SARS-CoV-2 as only part of the viral proteins are deleted, however it is crucial to define the attenuation mechanisms (19, 20). Pseudotyped viruses based only on the Spike protein, although they rule out the response against other viral antigens, allow the easy production of mutant S proteins, thus rapidly set up safe tests against new variants or introduce specific point mutation to evaluate the importance of selected protein regions (26).

In this context, we have set up a neutralization assay for SARS-CoV-2, producing ACE2/TMPRSS2-stably expressing target cell line and a pseudotyped lentiviral vector harboring the S protein of SARS-CoV-2 with detection based on luminescence.

The reliability of our assay was demonstrated in comparison to the MNT gold standard assay based on live virus, by testing residual anonymized samples negative to anti-SARS-CoV-2 antibodies (pre-pandemic and healthy unvaccinated individuals) and samples collected from COVID-19 convalescent individuals and vaccinees at different time ranges from the last dose administration. The PVNA assay showed a substantial agreement in the detection of nAbs as compared to the gold standard. We observed linear significant correlation of nAb levels (pVNT_90_ and MNT_90_) either with WT, Delta or Omicron VOC, both in convalescent individuals with natural response and vaccinees with different vaccine formulation. Lower sensitivity in samples with very low nAb MNT_90_ was observed for PVNA.

We report results of our test as 90% neutralization. This value was established in order to assess the new assay in relationship to the gold standard assay based on live virus, which is set for the estimation of 90% neutralization. As several research articles report neutralization assay with a threshold at 50% of neutralization, we also added as **Supplementary Data** the pVNT_50_ values. It should be noted that, while the GMT values for each group are higher, the overall results here discussed are unaffected, confirming the reliability of our assay.

In addition, as evidenced by the false positive result obtained, the PVNA could be limited by the potential interference of reverse transcription or integration of luciferase reporter gene in subjects on anti-retroviral therapy (15). For this reason, we added in the assay a control with VSVg pseudotyped lentivirus to exclude potential nAb false positive samples from the study.

The detailed analysis of samples collected from vaccinated individuals showed that vaccination with mRNA-1273 induced higher nAb levels both after short and long time from the second dose administration than vaccination with BNT16b2, AZD1222 and Ad26.CoV2.S vaccines. However, we have observed the absence of nAbs in Ad26 vaccinated individuals six months after the single administration. This is in contrast to the report published by GeurstvanKassel et al. (27) who analyzed nAb levels in health care workers (HCW) immunized with mRNA and viral vector vaccines showing their persistence although at a decreased level six months after completing the two dose vaccination with the different vaccine formulations, including one dose of Ad26.CoV2.S vaccines. However, it should be emphasized that our cohort was probably less exposed to virus contact with respect to HCWs.

Various reports have shown that sera isolated from vaccinated individuals have reduced neutralizing activity against VOCs as Delta and Omicron (28, 29). It has been speculated that vaccines inducing high level nAbs are still efficacious against VOCs where the Spike protein is similar to the WT. When titres are lower (due to vaccine type and VOC with cumulative spike mutations), small additional changes in nAb titres (age, immune status, waning of immune response) produce a stronger effect on vaccine efficacy (30). In our study, nAbs targeting the Delta and the Omicron variants were significantly lower in BNT16b2, mRNA-1273, and AZD1222 groups compared to antibodies targeting WT. However, samples with higher nAb levels showed a less decrease after long time from the completion of the vaccination schedule.

Finally, in agreement with previous studies (31, 32), we observed that a booster vaccination increase nAb titers against the Omicron variant. Importantly, here we report the persistence of neutralizing activity also against the Omicron VOC three month after the booster dose.

Overall, setting up a reliable neutralization assay easily performed in a not stringent containment remain a crucial issue and evidence are presented here on the fulfillment of these conditions by the analysis of sera of convalescent and vaccinated individuals, screening the ability of individual sera to neutralize VOCs after completed schedule of vaccinations and booster administration. This work may be also pivotal to standardize the various assays often applied in different laboratories in order to define the correlates of protection.

The study presented here has some limitations mainly due to the low sample size, the absence of a fixed time schedule of sample collection and the restricted, sometimes non-comparable, timing of the vaccination groups. This study was not designed to compare the effectiveness of the different vaccination strategies in generating an antibody response, but only to demonstrate that the proposed method is applicable and has a good analytical performance in multiple conditions, representing a valuable alternative for measuring nAb response. Moreover, the study is skewed toward healthy adult participants.

## Data availability statement

The raw data supporting the conclusions of this article will be made available by the authors, without undue reservation.

## Ethics statement

The studies involving human participants were reviewed and approved by Comitato Etico INMI Lazzaro Spallanzani IRCCS/Comitato Etico Unico Nazionale Covid-19” (issues n. 70/2018 and n. 55/2022). The patients/participants provided their written informed consent to participate in this study.

## Author contributions

LA provided plasmids. FC, GG, MF, GM, SM, DL, AB, KM developed and performed the MNT assay. LA, PDB, MT developed and performed the PVNA assay. FC, GG, LD’A, PDB, MT analyzed data. ADC, CC, FC, LD’A, PDB, MT conceived and supervised the studies. LD’A, PDB, MT wrote the original draft. ADC, FC, GG, LD’A, PDB, MT review and edit the draft. All authors read and approved the final manuscript.

## Funding

This study was supported by funds to the Istituto Nazionale per le Malattie Infettive (INMI) Lazzaro Spallanzani IRCCS, Rome (Italy), from Ministero della Salute (Ricerca Corrente - linea 1); the European Commission – Horizon 2020 (EU project 101003544 – CoNVat; EU project 101005075-KRONO) the European Virus Archive – GLOBAL (grants no. 653316 and no. 871029), and by funds from PON 2014-2020 “TITAN – Nanotecnologieper l’immunoterapia dei tumori” to CNR.

## Conflict of interest

Author LA was employed by company Takis Biotech.

The remaining authors declare that the research was conducted in the absence of any commercial or financial relationships that could be construed as a potential conflict of interest.

## Publisher’s note

All claims expressed in this article are solely those of the authors and do not necessarily represent those of their affiliated organizations, or those of the publisher, the editors and the reviewers. Any product that may be evaluated in this article, or claim that may be made by its manufacturer, is not guaranteed or endorsed by the publisher.
