# Supplementary material for: Comparative analysis of the neutralizing activity against SARS-CoV-2 Wuhan-Hu-1 strain and variants of concern: Performance evaluation of a pseudovirus-based neutralization assay
Source: Front Immunol. 2022 Sep 26;13:981693. doi: 10.3389/fimmu.2022.981693 (PMC9549111; doi:10.3389/fimmu.2022.981693)
Supplement: Supplementary file 1 [file Table_1.docx]

**Table s1.** Correlation between MNT_90_ and pVNT_90_ titers in serum samples from study participants.

|  |  |  |  |  | Spearman's Rank test ^a^ | |
| --- | --- | --- | --- | --- | --- | --- |
| Cohort | **Group** | ***n*** | ***n_tot_*** | **SARS-CoV-2 strain** | **r_s_** | ***p* value** |
| Study participants ^b^ | neg ctrl | 13 | 111 | WT | 0.9231 | < 0.0001 |
|  | post COVID-19 | 10 |  |  |  |  |
|  | short-time | 29 |  |  |  |  |
|  | long-time | 59 |  |  |  |  |
| Vaccinees  at ~1.5 mo | BNT162b2 | 9 | 29 | WT | 0.9139 | < 0.0001 |
|  | mRNA-1273 | 10 |  |  |  |  |
|  | AZD1222 | 10 |  |  |  |  |
| Vaccinees  at 4-6 mo | BNT162b2 | 14 | 59 | WT | 0.8444 | < 0.0001 |
|  | mRNA-1273 | 15 |  |  |  |  |
|  | AZD1222 | 15 |  |  |  |  |
|  | Ad26.COV2.S | 15 |  |  |  |  |
| Vaccinees  at ~1.5 mo | BNT162b2 | 9 | 29 | Delta | 0.9486 | < 0.0001 |
|  | mRNA-1273 | 10 |  |  |  |  |
|  | AZD1222 | 10 |  |  |  |  |
| Vaccinees  at 4-6 mo | BNT162b2 | 14 | 59 | Delta | 0.8844 | < 0.0001 |
|  | mRNA-1273 | 15 |  |  |  |  |
|  | AZD1222 | 15 |  |  |  |  |
|  | Ad26.COV2.S | 15 |  |  |  |  |
| Vaccinees  at ~1.5 mo | BNT162b2 | 9 | 29 | Omicron | 0.7341 | < 0.0001 |
|  | mRNA-1273 | 10 |  |  |  |  |
|  | AZD1222 | 10 |  |  |  |  |
| Vaccinees  at 4-6 mo | BNT162b2 | 14 | 59 | Omicron | 0.5376 | < 0.0001 |
|  | mRNA-1273 | 15 |  |  |  |  |
|  | AZD1222 | 15 |  |  |  |  |
|  | Ad26.COV2.S | 15 |  |  |  |  |

^a^ Spearman rank correlation was used as non-parametric test to measure the association between 90% live SARS-CoV-2 neutralization titers (MNT_90_) and 90% pseudovirus neutralization titers (pVNT_90_); ^b^ Study participants include: negative controls (pre-pandemic and unvaccinated not infected individuals); post-COVID-19 (convalescent individuals recovered from COVID-19); short-time (vaccinated individuals analysed at ~ 1.5 months from the second vaccine dose); and long-time (vaccinated individuals analysed at 4-6 months from the second vaccine dose). *n*: number of samples; *n_tot_*: total number of samples; r_s_: Spearman's Rank correlation coefficient; neg ctrl: negative controls; WT: wild type; mo: months.
