# Supplementary material for: Comparative analysis of the neutralizing activity against SARS-CoV-2 Wuhan-Hu-1 strain and variants of concern: Performance evaluation of a pseudovirus-based neutralization assay
Source: Front Immunol. 2022 Sep 26;13:981693. doi: 10.3389/fimmu.2022.981693 (PMC9549111; doi:10.3389/fimmu.2022.981693)
Supplement: Supplementary file 2 [file DataSheet_1.pdf]

Comparative analysis of the neutralizing activity against SARS-CoV-2 Wuhan-Hu-1 strain and variants of concern: performance evaluation of a pseudovirus-based neutralization assay

Supplementary methods

Study design and participants

Negative samples for anti-SARS-CoV-2 antibodies included samples collected in early 2019 (named as pre-pandemic samples, n=10) and samples from unvaccinated not infected individuals (n=4) and were tested to evaluate the specificity of PVNA. Samples positive for anti-Spike Receptor Binding Domain (RBD) antibodies included sera collected from convalescent individuals recovered from COVID-19 after a median time of 93 days (interquartile range, IRQ=84.5-99; n=10) and sera from vaccinated individuals (n=89). These samples were used to evaluate the presence of nAbs and compare the titers between the two assays. As shown in **Fig. 1a**, for vaccinees' samples two different time points from the last vaccine dose administration were considered, "short-time" (n=29) consisting in samples collected after an overall median time of 47 days (IQR=32-73; ~ 1.5 months (mo)) after second vaccine dose and "long-time" (n=60) consisting in samples collected after an overall median time of 135 days (IQR=128-154; 4-6 mo) after the last vaccine dose administration. Indeed, in order to perform the PVNA evaluation on a representative group of population for the different COVID-19 vaccine formulations in use, vaccinees' group included: individuals vaccinated with two-doses of mRNA vaccines, both BNT162b2 (BioNTech, Pfizer, n=24) and mRNA-1273 (Moderna, n=25), with two-doses of chimpanzee adenovirus vector vaccine, AZD1222 (AstraZeneca, n=25) and with single-dose of human adenovirus vector vaccine, Ad26.COV2.S (Johnson & Johnson, n=15). Short-time and long-time samples were tested for BNT162b2 (n=9 and n=15, respectively), mRNA-1273 (n=10 and n=15, respectively), and AZD1222 (n=10 and n=15, respectively) groups, while long-time (n=15) samples only were analyzed for Ad26.COV2.S vaccine group.

Longitudinal samples collected from mRNA recipients (n=18) after a median time of 26 (IQR:17–29.8; ~1 mo) and 96 (IQR:86–102.8; ~3 mo) days from the third vaccine dose were also tested.

Plasmids

The pMD2.G plasmid encoding the spike glycoprotein of the vesicular stomatitis virus (VSV-G), the lentiviral transfer pTY2-CMV-LucWPRE plasmid coding for the Luciferase (Luc) reporter, and the lentiviral packaging plasmid pCMVΔR8.2 were kindly gifted from Dr. Andrea Cara (Istituto Superiore di Sanità, Rome, Italy). The lentiviral packaging plasmid psPAX2 was kindly gifted from Dr. Oreste Acuto (University of Oxford, Oxford, UK). The lentiviral transfer pLVX-IRES-Neo vector for the bicistronic expression of the transgene with the selection marker neomycin (Neo; G418 selection) was obtained from Clontech (https://www.snapgene.com/resources/plasmid-files/?set=viral_expression_and_packaging_vectors&plasmid=pLVX-IRES-Neo). The pmR-mCherry vector was kindly gifted from Dr. Dario Acampora (Institute of Genetics and Biophysics, CNR, Naples, Italy). The pCMV6-AC-TMPRSS2 expression vector encoding the human transmembrane serine protease 2 (hTMPRSS2) transcript variant 2 (RefSeq NM_005656.2, NP_005647.2) was obtained from OriGene (https://www.origene.com/catalog/cdna-clones/expression-plasmids/sc323858/tmprss2-nm_005656-human-untagged-clone). The full-length codon-optimized sequence of the human angiotensin-converting enzyme 2 (hACE2) (NM_021804.2; MC_0101086) in pcDNA3.1+/C-(K)-DYK expression plasmid carrying a C-terminal FLAG-tag (DYKDDDDK) was purchased from GenScript (pcDNA3.1-hACE2). The full-length codon-optimized sequence of the S protein of SARS-CoV-2 isolate Wuhan-Hu-1 (MC_0101081) was obtained by GenScript and subcloned into pcDNA3.1+/C-(K)-DYK (pcDNA3.1-2019-nCoV). 5’-CGCGGATCCGCCACCATGTTCGTCTTC-3’ and 5’-CCGGAATTCAACAGCAGGAGCCACAGC-3’ primers were used to amplify a truncated S sequence devoid of the endoplasmic reticulum (ER) retrieval signal consisting of the last 19 amino

acids (hereinafter referred to as SΔ19). Then, the truncated SΔ19 sequence was cloned into pcDNA4/V5-His A vector, generating the pcDNA4-SΔ19 plasmid.

hACE2 and hTMPRSS2 were subcloned into pLVX-IRES-Neo vector to generate pLVX-hACE2-IRES-Neo and pLVX-hTMPRSS2-IRES-Neo plasmids. mCherry was then subcloned into pLVX-hACE2-IRES vector after removing the Neo sequence *via* BstXI-MluI restriction digestion to generate pLVX-hACE2-IRES-mCherry. Plasmid encoding codon-optimized SARS-CoV-2 SΔ19 B.1.617.2 variant was synthetized and cloned by ThermoFisher into the pcDNA3.1 expression vector to generate pcDNA3.1-SΔ19Delta plasmid. The full-length codon-optimized sequence of the S protein of SARS-CoV-2 B.1.1.529 variant was obtained by GenScript and subcloned into pTK1A-Spike Omicron BA.1 II plasmid. Then, the truncated SΔ19Omicron sequence was cloned into pcDNA4/V5-His A vector to generate the pcDNA4-SΔ19Omicron plasmid.

Cell lines

HEK293 cell line was kindly provided by Dr. Valerio Costa (Institute of Genetics and Biophysics, CNR, Naples, Italy). HEK293T cell line was obtained from Clontech. HEK293, HEK293T, and HEK293-hACE2-hTMPRSS2 cells stably expressing ACE2/TMPRSS2 proteins were grown and maintained in humidified incubator at 37°C with 5% CO2 in Dulbecco's Modified Eagle Medium (DMEM) supplemented with high glucose, 25U/mL penicillin G, 25µg/mL streptomycin, 1% 100X L-Glutamine, and 10% fetal bovine serum (FBS) (GIBCO). For neutralization assays medium supplemented with 2% FBS was used. HEK293-hACE2-hTMPRSS2 cells were selected with 500µg/mL G418.

Generation of stable HEK293-hACE2-hTMPRSS2 cells

HEK293 cells stably expressing ACE2 together with TMPRSS2 were produced by lentiviral transduction. Briefly, 8µg of pLVX-hACE2-IRES-mCherry, 4µg of psPAX2, and 4µg of pMD2.G plasmids were co-transfected in HEK293T cells (seeded 24 hours before transfection at a density of

3.5×10^6 cells/100 mm Petri dish), using the PEIpro transfection reagent (Polyplus) following manufacturer's protocols to generate VSV-G pseudotyped lentiviral particles. Cell culture supernatant containing pseudoviruses was collected 72 hours after transfection, filtered (0.45 µm pore size filter) and used for transducing HEK293 cells. 48 hours after transduction, mCherry expression was evaluated by flow cytometry and hACE2-mCherry$^+$ HEK293 cells were sorted by FACS (FACSAria III, BD). 8µg of pLVX-TMPRSS2-IRES-Neo, 4µg of psPAX2 and 4µg of pMD2.G plasmids were used to produce in HEK293T cells VSV-G pseudotyped lentiviral particles harboring the TMPRSS2 gene. Cell culture supernatants containing pseudovirions were collected 72 hours after transfection, filtered (0.45 µm pore size filter) and used to transduce sorted ACE2-mCherry$^+$ HEK293 cells for generating HEK293-hACE2-hTMPRSS2 cells. 48 hours after transduction, G418 (500ug/mL) was added to the culture to select transduced hACE2-mCherry$^+$/TMPRSS2-Neo$^+$ HEK293 cells by Neo expression. Whole cell lysates from ACE2$^+$ HEK293 cells were run on SDS-PAGE and probed with anti-FLAG recombinant rabbit polyclonal antibody (Thermo Fisher Scientific, 1/1000) and goat anti-rabbit IgG (whole molecule)-Peroxidase (Sigma-Aldrich, 1/10000) secondary antibody to confirm ACE2 expression by Western blot. Polyclonal anti-TMPRSS2 primary antibody (Thermo Fisher Scientific, 2 mg/mL) and AF633 goat anti-rabbit IgG (Thermo Fisher Scientific, 2 mg/mL) secondary antibody were used to detect cell surface expression of TMPRSS2 by FACS.

Production and titration of SARS-CoV-2 S-pseudotyped lentiviruses

8µg of pTY2-CMV-Luc$_{WPRE}$, 4µg of pCMVΔR8.2 and 4µg of pcDNA4-SΔ19 or pcDNA3.1-SΔ19Delta or pcDNA4-SΔ19Omicron plasmids were co-transfected on 3.5×10^6 HEK293T cells using the PEIpro transfection reagent following manufacturer's protocols. Cell culture supernatants containing pseudovirions were collected 72 hours after transfection, filtered (0.45 µm pore size filter), aliquoted and stored at -80°C until use. 8µg of pTY2-CMV-Luc$_{WPRE}$, 4µg of pCMVΔR8.2 and 4µg of VSVg plasmids were used to produce VSVg pseudotyped lentivirus.

Pseudovirus titers were determined according to Neerukonda et al. [15]. In detail, HEK293-hACE2-hTMPRSS2 target cells (1.0×10^4/well) were plated into 96-well plates 24 hours before the pseudoviral infection. The following day, 50 µl of undiluted or twofold serially diluted pseudovirus-containing cell culture supernatants (ranging from 1/2 to 1/128) were added to target cells. 72 hours later, cells were harvested and lysed using the Steady Glo luciferase assay system (Promega) to measure luciferase activity by Victor multilabel plate reader (Perkin Elmer). Pseudovirus titers were expressed as relative luminescence units (RLUs) per mL of pseudovirus supernatants (RLU/mL).

Neutralization assay was performed according to Ni et al. [16]. Briefly, 50 µl of SARS-CoV-2 S-pseudotyped (Wuhan-Hu-1 isolate, B.1.617.2 or B.1.1.529 variants) lentiviruses (1×10^6 RLU/mL) were mixed with 50 µl of twofold serially diluted heat-inactivated serum samples (ranging from 1/10 to 1/640) at 37°C for 1 hour. The mixtures (100 µl) were then transferred to 96-well plates seeded with HEK293-hACE2-hTMPRSS2 target cells (1.0×10^4/well) 24 hours before the pseudoviral infection. 72 hours later, cells were harvested and lysed using the Steady Glo luciferase assay system (Promega) to measure the luciferase activity by Victor multilabel plate reader. All samples were run in triplicate.

Supplementary Figure Legends

Figure s1. Anti-RBD antibody detection. Anti-Spike/RBD IgG quantitative detection in sera collected at a median time of (**a**) ~ 1.5 month (mo) post-second vaccine dose, (**b**) ~ 4–6 months post-vaccination. Graphs show median values. Kruskal–Wallis test with Dunn's multiple comparison post-test was used to compare groups. * $p < 0.05$; ** $p \leq 0.01$; **** $p < 0.0001$. BAU: Binding Antibody Units. *GMT*: geometric mean titers; n = number of samples.

Figure s2. MNT_{90} and $pVNT_{90}$ values strongly correlate with anti-RBD IgG. Correlation between MNT_{90} and anti-RBD in sera collected at (**a**) ~ 1.5 mo post second dose or (**b**) ~ 4–6 months post-vaccination. Correlation between $pVNT_{90}$ and anti-RBD in sera collected at (**c**) ~ 1.5 mo post-second dose or (**d**) ~ 4–6 months post-vaccination. Spearman rank correlation was performed. The non-parametric Spearman's correlation coefficients (r_s) and statistically significant p values were provided. Perfect-fit correlation line was included on the plots.

Figure s3. PVNA showing SARS-CoV-2 WT neutralization at a threshold of 50%. (**a–c**) 50% pseudovirus neutralization titers using pseudotyped lentiviruses carrying the WT SΔ19 protein ($pVNT_{50}$) were used as measure of nAb levels in sera from (**a**) all study participants; (**b**) unvaccinated and recipients of two-dose BNT162b2, mRNA-1273, and AZD1222 collected at a median time of ~ 1.5 mo post-second vaccine dose; (**c**) unvaccinated and recipients of two-dose BNT162b2, mRNA-1273, AZD1222, or one dose Ad26.CO2.S collected at a median time of ~ 4–6 mo post-vaccination. Geometric means ± 95% confidence intervals were reported. Neutralization titers < 10 were considered negative and given an arbitrary value of 5 (dotted line). Kruskal–Wallis test with Dunn's multiple comparison post-test was used to compare groups. * $p < 0.05$; ** $p \leq 0.01$; *** $p \leq 0.001$; **** $p < 0.0001$.

Figure s4. PVNA showing SARS-CoV-2 Delta neutralization at a threshold of 50%. (**a–b**) 50% pseudovirus neutralization titers using pseudotyped lentiviruses carrying the Delta SΔ19 protein ($pVNT_{50}$) were used as measure of nAb levels in sera from (**a**) unvaccinated and recipients of two-dose BNT162b2, mRNA-1273, and AZD1222 collected at a median time of ~ 1.5 mo post-second vaccine dose; (**b**) unvaccinated and recipients of two-dose BNT162b2, mRNA-1273, AZD1222, or one dose Ad26.CO2.S collected at a median time of ~ 4–6 mo post-vaccination. Geometric means ± 95% confidence intervals were reported. Neutralization titers < 10 were considered negative and given an arbitrary value of 5 (dotted line). Kruskal–Wallis test with Dunn's multiple comparison post-test was used to compare groups. * $p < 0.05$; ** $p \leq 0.01$; *** $p \leq 0.001$; **** $p < 0.0001$.

Figure s5. Vaccine-induced nAbs are less effective against Delta variant. $pVNT_{90}$ against SARS-CoV-2 pseudotyped lentiviruses carrying the WT or the Delta SΔ19 protein in sera from each individual collected at ~ 1.5 mo post-second dose of (**a**) BNT162b2, (**c**) mRNA-1273, (**e**) AZD1222 or at ~ 4–6 months post-vaccination with (**b**) BNT162b2, (**d**) mRNA-1273, (**f**) AZD1222 or (**g**) Ad26.CO2.S were reported. A two-tailed non-parametric Wilcoxon signed-rank test was performed for paired observations. * $p < 0.05$; *** $p \leq 0.001$. Geometric mean titers are reported. n = number of samples.

Figure s6. PVNA showing SARS-CoV-2 Omicron neutralization at a threshold of 50%. (**a–c**) 50% pseudovirus neutralization titers using pseudotyped lentiviruses carrying the Omicron SΔ19 protein ($pVNT_{50}$) were used as measure of nAb levels in sera from (**a**) unvaccinated and recipients of two-dose BNT162b2, mRNA-1273, and AZD1222 collected at a median time of ~ 1.5 mo post-second vaccine dose; (**b**) unvaccinated and recipients of two-dose BNT162b2, mRNA-1273, AZD1222, or one dose Ad26.CO2.S collected at a median time of ~ 4–6 mo post-vaccination; (**c**) unvaccinated and SARS-CoV-2 infected recovered donors. Geometric means ± 95% confidence

intervals were reported. Neutralization titers < 10 were considered negative and given an arbitrary value of 5 (dotted line). Kruskal–Wallis test with Dunn's multiple comparison post-test was used to compare vaccine groups. * $p < 0.05$. A two-tailed non-parametric Mann–Whitney test was used to compare unvaccinated and COVID-19 convalescents.

Figure s7. Vaccine-induced nAb response is weak against Omicron VOC. $pVNT_{90}$ against SARS-CoV-2 pseudotyped lentiviruses carrying the WT or the Omicron SΔ19 protein in sera from each individual collected at ~ 1.5 mo post second dose of (**a**) BNT162b2, (**c**) mRNA-1273, (**e**) AZD1222 or at ~ 4–6 months post-vaccination with (**b**) BNT162b2, (**d**) mRNA-1273, (**f**) AZD1222 or (**g**) Ad26.CO2.S were reported. A two-tailed non-parametric Wilcoxon signed-rank test was performed for paired observations. * $p < 0.05$; ** $p \leq 0.01$; *** $p \leq 0.001$. Geometric mean titers are reported. n = number of samples.

Figure s8. Booster response analysis showing neutralization against SARS-CoV-2 WT and Omicron variant at threshold of 50%. (**a**–**b**) Violin plots representing Log of $pVNT_{50}$ observed in sera ($n = 15$) collected at ~ 1 mo or ~ 3 mo after vaccine boost and challenged with (**a**) SARS-CoV-2 WT or (**b**) Omicron pseudotyped lentiviruses. A two-tailed non-parametric Mann–Whitney test for unpaired observations was performed. * $p < 0.05$. n = number of samples.
