# Supplementary figures and images for: Comparative analysis of the neutralizing activity against SARS-CoV-2 Wuhan-Hu-1 strain and variants of concern: Performance evaluation of a pseudovirus-based neutralization assay
Source: Front Immunol. 2022 Sep 26;13:981693. doi: 10.3389/fimmu.2022.981693 (PMC9549111; doi:10.3389/fimmu.2022.981693)

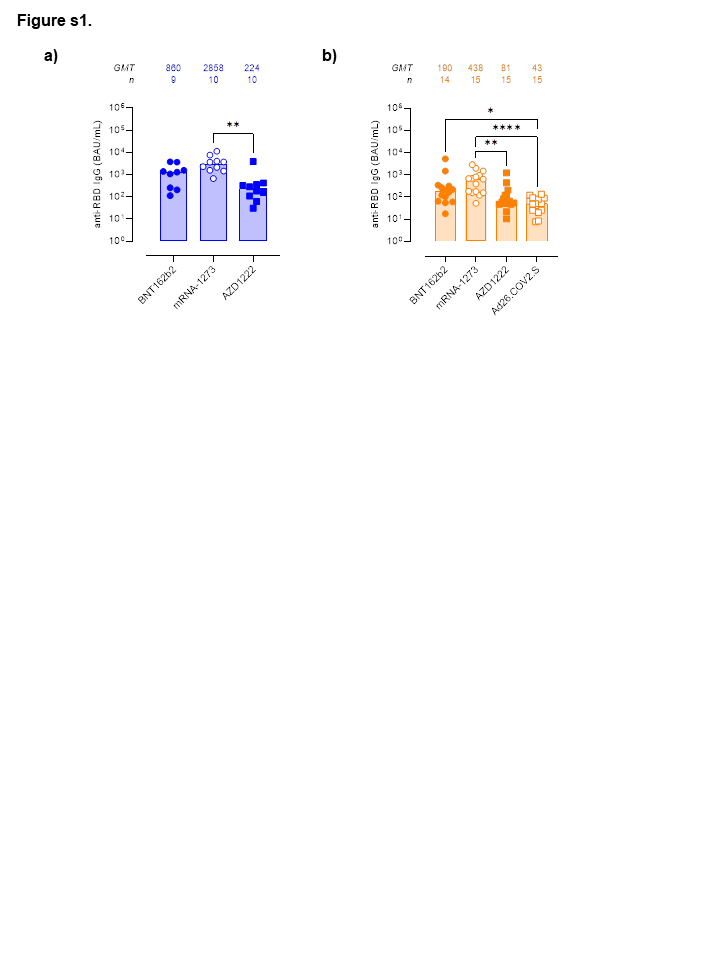

Supplement: Supplementary file 3 [file Image_1.tif]

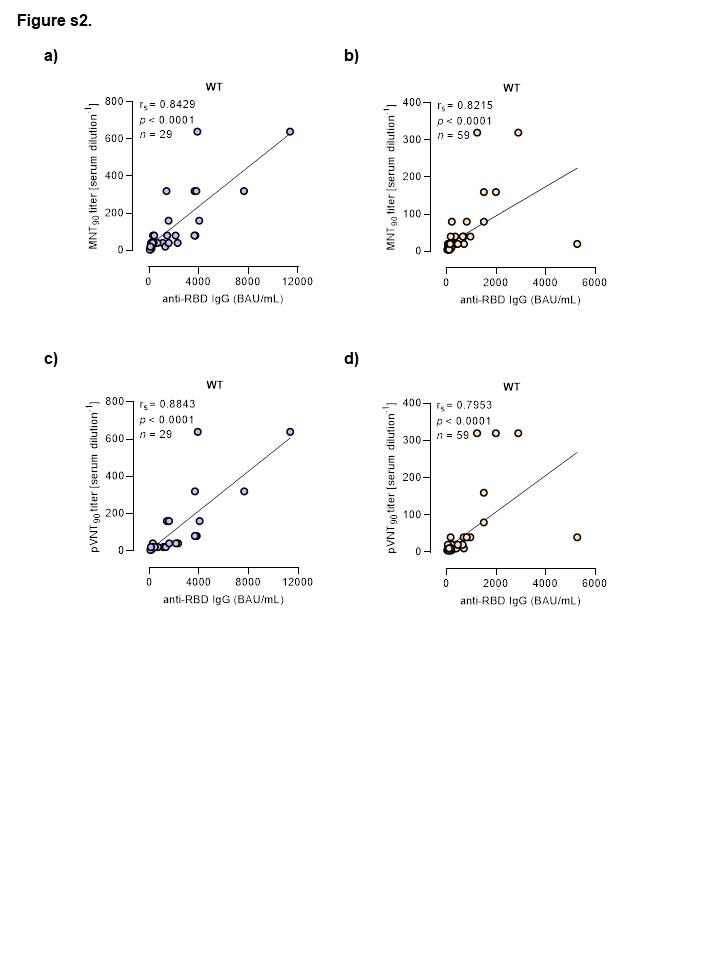

Supplement: Supplementary file 4 [file Image_2.tif]

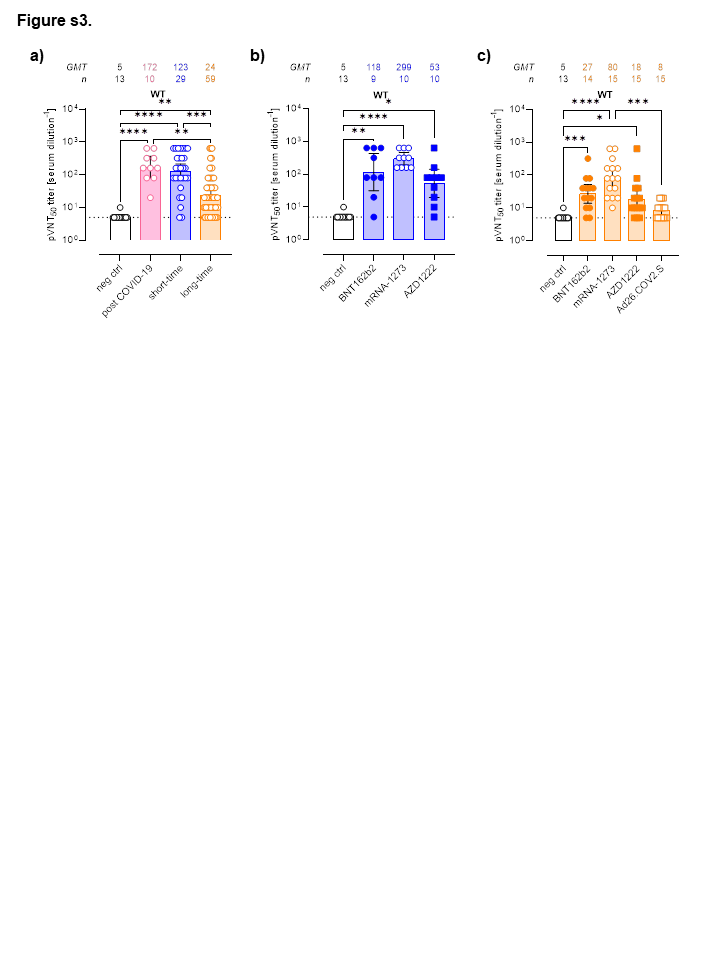

Supplement: Supplementary file 5 [file Image_3.tif]

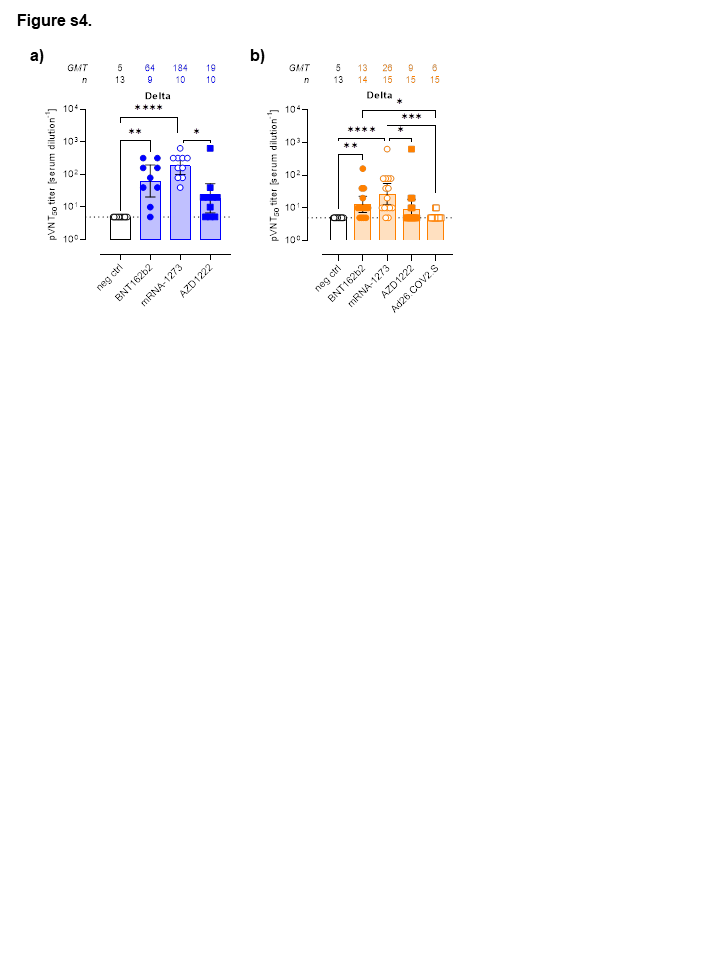

Supplement: Supplementary file 6 [file Image_4.tif]

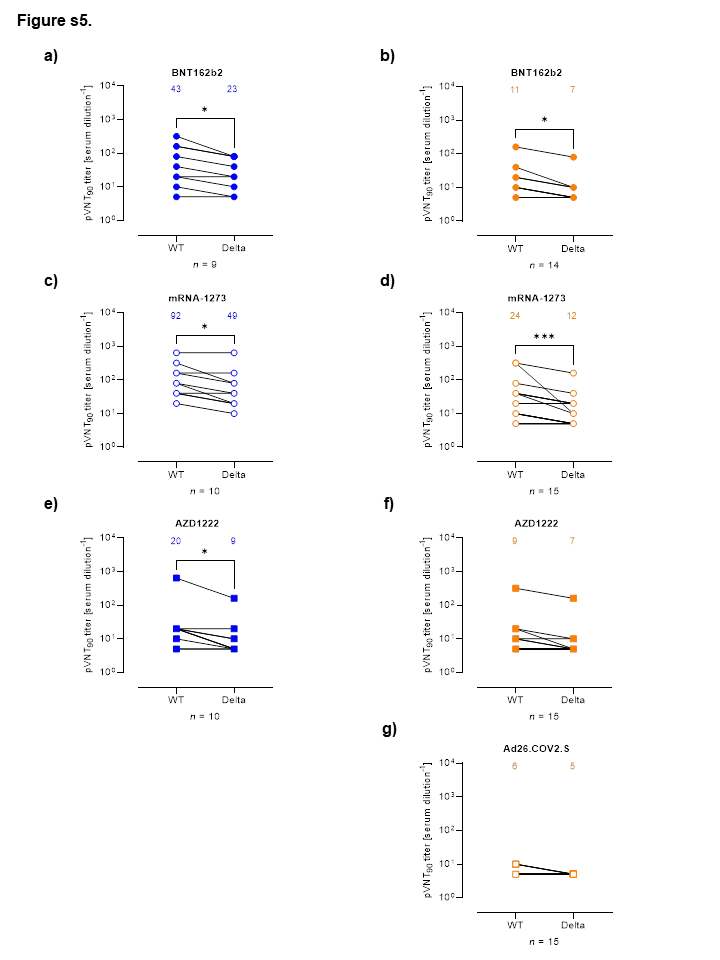

Supplement: Supplementary file 7 [file Image_5.tif]

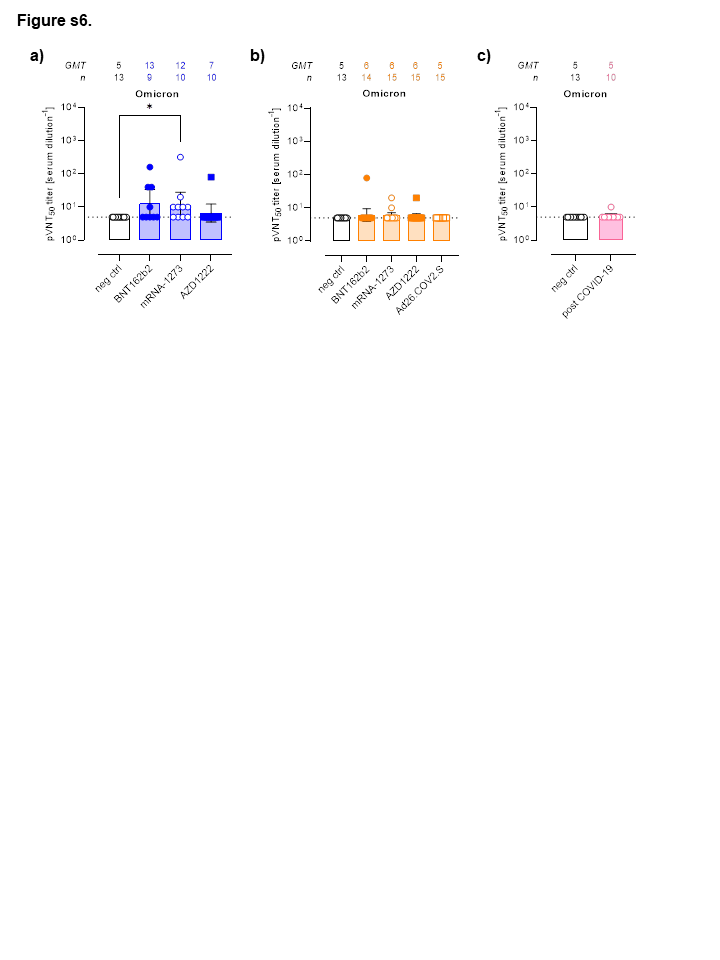

Supplement: Supplementary file 8 [file Image_6.tif]

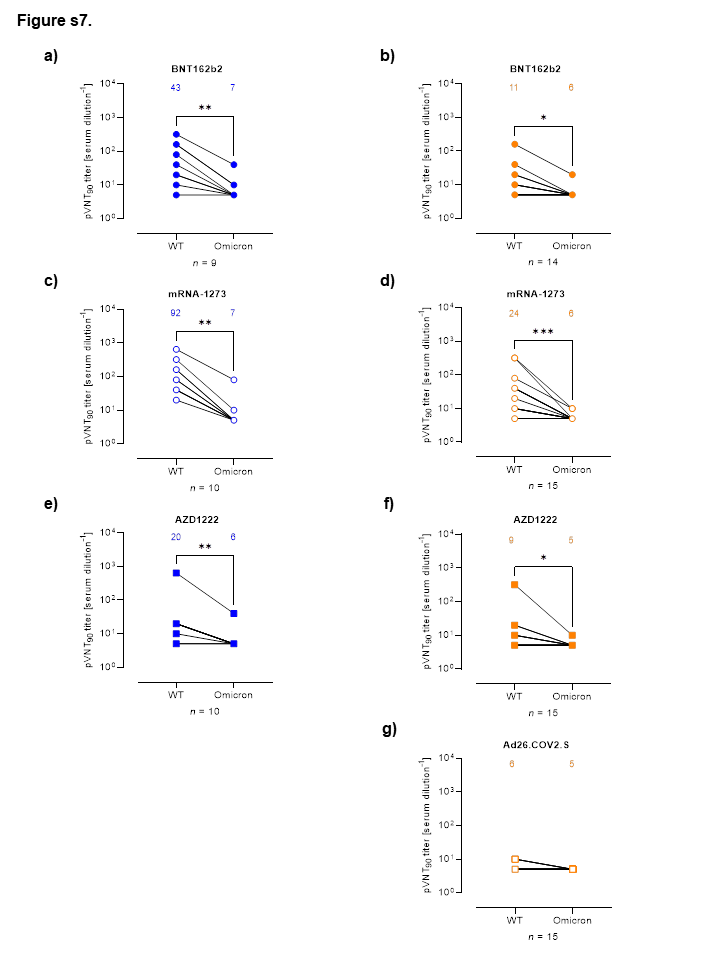

Supplement: Supplementary file 9 [file Image_7.tif]

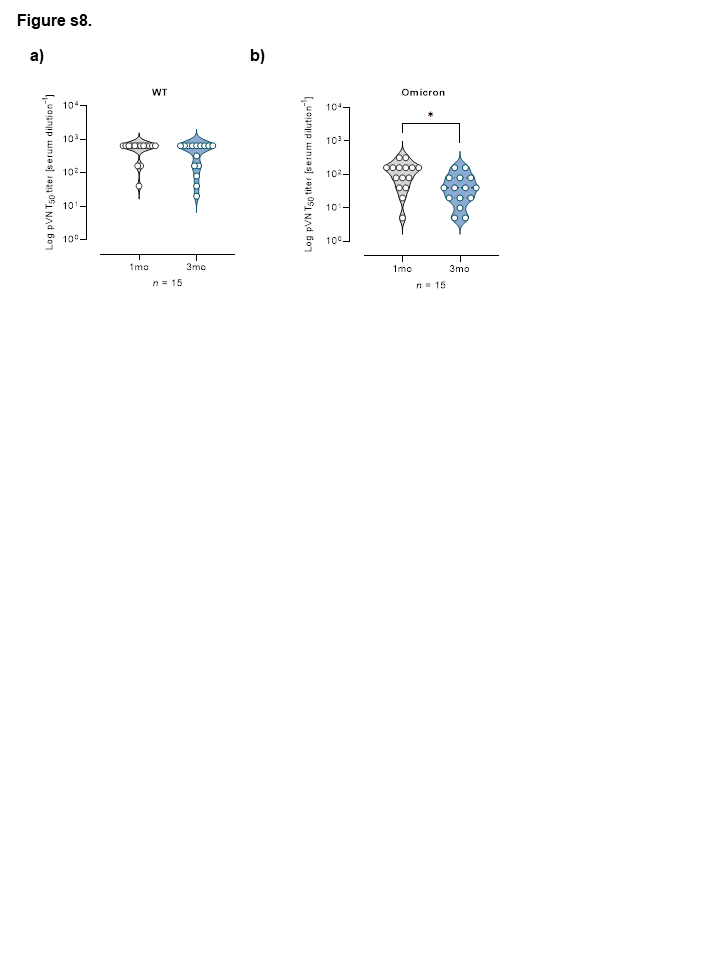

Supplement: Supplementary file 10 [file Image_8.tif]
